# 16th Carbonyl Metabolism Meeting: from enzymology to genomics

**DOI:** 10.1186/1479-7364-6-25

**Published:** 2012-12-01

**Authors:** Edmund Maser

**Affiliations:** 1 Institute of Toxicology and Pharmacology for Natural Scientists, University Medical School, Schleswig-Holstein, Campus Kiel, Brunswiker Str. 10, Kiel, D-24105, Germany

**Keywords:** Carbonyl metabolism, Alcohol dehydrogenase (ADH), Aldehyde dehydrogenase (ALDH), Medium-chain dehydrogenase (MDR), Short-chain dehydrogenase/reductase (SDR), Aldo-keto reductase (AKR)

## Abstract

The 16th International Meeting on the Enzymology and Molecular Biology of Carbonyl Metabolism, Castle of Ploen (Schleswig-Holstein, Germany), July 10–15, 2012, covered all aspects of NAD(P)-dependent oxido-reductases that are involved in the general metabolism of xenobiotic and physiological carbonyl compounds. Starting 30 years ago with enzyme purification, structure elucidation and enzyme kinetics, the Carbonyl Society members have meanwhile established internationally recognized enzyme nomenclature systems and now consider aspects of enzyme genomics and enzyme evolution along with their roles in diseases. The 16th international meeting included lectures from international speakers from all over the world.

## Background

The International Meeting on the Enzymology and Molecular Biology of Carbonyl Metabolism was established 30 years ago by Prof. Dr. Henry Weiner, Purdue University, West Lafayette, IN, USA, and was later co-organized by his wife Esther. Starting in 1982 in Bern, Switzerland, the meeting was held every other year, alternately in USA and elsewhere in the world. This has been the recurring plan until today. At these meetings, talks and posters are presented on oxido-reductases that use a carbonyl as a substrate: alcohol dehydrogenases (ADH), aldehyde dehydrogenases (ALDH), short-chain dehydrogenases/reductases (SDR) and aldo-keto reductases (AKR). Previously, topics ranged from biochemical enzymology to gene regulation and the function of the enzyme in the cell. Important aspects of molecular toxicology were introduced in parallel, such as the deleterious effects of lipid-derived reactive aldehydes and their metabolic detoxification. Later, important aspects on enzyme genomics and enzyme evolution were included and now cover eukaryotic as well as prokaryotic organisms. This is why this meeting brings so many interested and engaged scientists from various disciplines together and has such a great tradition and tremendous success.

The aim of the meeting is not only to provide timely updates on all aspects within the field of carbonyl metabolism to the senior scientists and to attract new investigators, but also to educate and train younger scientists and to provide them an opportunity to discuss with senior scientists in a relaxed atmosphere. Excellent personal interactions during the meeting have resulted in a number of international collaborations that emerged among the participants and were very important for the advancement of science in the field of carbonyl metabolism.

Each of the previous 15 meetings was a unique experience for the participants. Moreover, the half-day social excursions have become one of the most anticipated events at each meeting.

The scientific papers of each meeting were published in the form of a book under the editorship of Henry Weiner and the help of three co-editors, who ensured a review process that meets the criteria of international peer-reviewed journals. Later, in 2001, 2003, 2009 and 2011, the manuscripts of every volume appeared as a special issue of the international journal *Chemico*-*Biological Interactions*. The articles represent a permanent record of what was presented during the conferences and provide a view on the continuous scientific progress achieved on the carbonyl metabolizing enzyme super-families within the last 30 years.

### The 16th meeting on the enzymology and molecular biology of carbonyl metabolism

The 16th meeting was no exception. Sixteen countries were represented, with 53% of participants coming from Europe, 32% from North America and 15% from Asia and Australia. This year's program was put together by Hans-Joerg Martin and Edmund Maser. Over 70 talks and posters provided the participants with a wide variety of presentations dealing with enzymology, molecular biology and metabolic aspects of carbonyl metabolizing oxido-reductases. Much new information was presented, including three dimensional structures of enzymes not previously reported, new aspects of gene regulation, and metabolism and enzyme mechanisms, as well as enzyme genomics and evolution along with sequence alignments. In addition, there were nomenclature updates for the different enzyme super-families. Importantly, there was an increased emphasis and information pertaining to the emerging physiological/pathological roles and significance of the carbonyl-metabolizing enzymes. Specifically, several new milestone findings were associated to human diseases, and the development of respective medical treatment strategies was discussed. The level of the science presented was top notch, a fact that was recognized by a speaker from the pharmaceutical industry. The full program can be seen on the homepage of the meeting: http://www.carbonyl.toxi.uni-kiel.de/.

A new feature was the competition for the best poster presentation by pre-doctoral and post-doctoral researchers. Members of the Poster Award Committee reviewed the posters and selected two finalists who shared the ‘Henry and Esther Weiner Award 2012’ which was named after the founder and his wife of the carbonyl metabolism study group.

### Details of the scientific programme

The conference was kicked off by Edmund Maser (Kiel University Medical School, Germany) who first welcomed all the participants and then gave a brief overview on the 30 years' history of the Carbonyl Metabolism meetings. He also summarized several recent hot spots in carbonyl metabolism research.

#### ALDH session

Vasilis Vasiliou (University of Colorado, USA) opened the scientific section on the super-family of ALDH with an introduction into ALDH evolution. He gave an update on the enzymes' systematic nomenclature and summarized recent findings on the physiological and pathophysiological role of these enzymes in humans and other organisms.

Lilian Gonzalez-Segura (Universidad Nacional Autónoma de México, México) continued by discussing the structural, phylogenetic and functional evidence for the existence of specific potassium-binding sites in ALDHs. Based on her results, she concluded that most of the ALDH enzymes possess intra-subunit sites, few have inter-subunit sites, and only the ALDH9s from *Pseudomonas* spp. have central-cavity sites.

Roger Holmes (Griffith University, Australia) focused on the comparative genomics and proteomics of ALDH2 and ALDH1B1, which are both mitochondrial enzymes that metabolize acetaldehyde and other biological aldehydes in the human body. He proposed that a dominant negative heterodimerization of ALDH2*2 subunits with ALDH1B1 may partially explain a lack of compensation by ALDH1B1 in ALDH2*2 individuals and that the *ALDH1B1* gene originated in early vertebrates from a retro-transposition of the *ALDH2* gene.

Tom Hurley (Indiana University School of Medicine, USA) gave a perspective on the discovery of selective and non-selective inhibitors of ALDH1A1 and ALDH3A1 to overcome resistance towards cyclophosphamide upon chemotherapy. He and his group have identified new compounds which will be further developed in different cell line studies.

Sergey Krupenko (Medical University of South Carolina, USA) reported on an unusual mode of coenzyme binding in the carboxy-terminal domain of ALDH1L1 which is a natural fusion of three unrelated genes and catalyzes the two-step conversion of 10-formyltetrahydrofolate to tetrahydrofolate and CO_2_ with NADP^+^ as cofactor. A variety of amino acid substitutions led him to conclude that Glu673 restricts the affinity for the co-factor, whereas Cys707 acts as the sensor of the co-factor redox state.

In her talk, Rosario Muñoz-Clares (Universidad Nacional Autónoma de México, México) focused on the structural and functional aspects of plant ALDH10 enzymes and their role in glycine betaine (GB) production by oxidation of betaine aldehyde (BAL). Biochemical and phylogenetic analyses support the existence of two kinds of ALDH10 isozymes: those with low-BAL affinity, present in most plants, and those with high-BAL affinity, only present in GB-accumulator plants.

Naim Stiti (University of Bonn, Germany) discussed the role of ALDHs in abiotic stress and the detoxification of reactive aldehydes derived from lipid peroxidation in *Arabidopsis thaliana*. He found that the enzyme activities were redox-dependent and that thiol groups of redox-sensitive cysteines at the surface of the protein subunits are critical to dimerization and inactivation.

Vasilis Vasiliou (University of Colorado, USA) introduced a novel concept of an ALDH being involved in gout, a common form of inflammatory arthritis that results from hyperuricemia. He discussed the structural determinants of ALDH16A1 during evolution and postulated that ALDH16A1 may interact with several other proteins associated with uric-acid formation, diabetes, vesicular transport and protein degradation.

François Talfournier (CNRS-Université de Lorraine, France) focused on key roles played by the structural dynamics associated with the co-factor binding in the catalytic mechanism of ALDHs. Structural analyses together with kinetic data from amino acid substitutions at critical residues led him to hypothesize that the co-factor binding mode is in part responsible for the different kinetic behaviour of hydrolytic and CoA-dependent ALDHs.

Shih-Jiun Yin (National Defense Medical Center, Taiwan) presented his work on the expression pattern, activities and protein contents of ADHs and ALDHs in human liver. He emphasized that functional allelic variations at the *ADH1B* and *ALDH2* gene loci affect the development of alcoholism and may be involved in the pathogenesis of alcohol-related liver diseases.

#### ADH session

Hans Jörnvall (Karolinska Institutet Stockholm, Sweden) opened the scientific section on the ADH and highlighted the origin and evolution of the super-families of medium-chain alcohol dehydrogenases/reductases (MDR) and SDR. He concluded that ADHs have a common origin, with at least three separate emergences, the SDRs, the metal-free MDRs and the Zn-dependent MDRs, from the common ancestor.

Hector Riveros-Rosas (Universidad Nacional Autónoma, México) continued with evolutionary insights on the metabolic role developed by ADHs in animals. He claimed that the presence of ADHs in vertebrates has not been a consequence of chronic ethanol exposure and that their participation in ethanol metabolism can be considered incidental, and not adaptive.

Bryce Plapp (University of Iowa, USA) described the biochemical basis for yeast strains that are fitter for growth in the presence of allyl alcohol. He and his coworkers did site-directed mutagenesis at critical sites in ADH1 and determined steady-state kinetic constants. They found that the fitter yeasts are ‘bradytrophs’ (slow growing) under fermentative conditions because the ADHs have decreased catalytic efficiency and produce less of the toxic acrolein.

Jan-Olov Höög (Karolinska Institutet Stockholm, Sweden) addressed the yet unknown function of mammalian ADH5, which so far could not be isolated as native protein. Computational methods including sequence alignments, homology modelling and molecular dynamics implied that the protein does not fold properly, is not stable, is regulated on the mRNA level, or might even be a pseudogene.

Stephanie MacAllister (University of Toronto, Canada) presented her research comparing the toxicity mechanisms of acrolein and chloroacetaldehyde (CAA) which are derived from the metabolism of the anti-cancer drugs ifosfamide and cyclophosphamide. By using a variety of model systems, they found that acrolein was significantly more toxic than CAA, and that thiol groups, such as *N*-acetylcysteine and cysteine, were the most effective protective agents against both acrolein and CAA biomarker toxicities.

David Kopecny (Palacky University, Czech Republic) presented a structure-functional study on *S*-nitrosoglutathione reductase from tomato. This enzyme is also known as *S*-(hydroxymethyl)glutathione dehydrogenase, which belongs to the ADH3 family branch of the ADH super-family.

#### AKR/SDR session

Edmund Maser (Kiel University Medical School, Germany) opened the scientific session on the AKR and SDR superfamilies with a brief introduction into their history. He then gave a perspective on important events during the evolution of these two super-families and highlighted the association of important AKR and SDR to disease including diabetes, hypertension, the metabolic syndrome, osteoporosis and cancer.

Bengt Persson (Linköping University, Sweden) gave a talk on the nomenclature of the SDR and explained the sub-division of this large super-family using bio-informatics methods. In his opinion, identification of new SDR families is slowing down, even though the number of SDR enzymes increases continuously, indicating that the classification system works and that we will know all SDR families in a not-too-distant future.

Natalia Kedishvili (University of Alabama, Birmingham, USA) focused on the role of the SDR16C family of proteins in retinoic acid homeostasis, including SDR16C4 (retinol dehydrogenase 10), which is indispensable for retinoic acid biosynthesis during vertebrate embryogenesis, SDR16C1, SDR16C5 and SDR16C6. She concluded that the SDR16C family is essential in the regulation of retinoic acid biosynthesis during embryogenesis and possibly in adulthood.

Xavier Parés (Universitat Autònoma de Barcelona, Spain) first reviewed the role of cytosolic MDR and membrane-bound SDR in retinoid metabolism. Then, he reported on the identification of cytosolic AKR1C3 and AKR1B10 with retinaldehyde reductase activity, the latter having a possible relevance in cancer. He suggested that retinoid analogues could be a good starting point for searching AKR1B10-selective inhibitors.

Jaume Farrés (Universitat Autònoma de Barcelona, Spain) continued with his data on AKR1C3 as a highly efficient 9-cis-retinaldehyde reductase which is elevated in different cancer types and is also involved in chemotherapeutic-drug resistance. Farrés could show that the proliferative action of AKR1C3 involves the retinoic acid signalling pathway and that this is in part due to the retinaldehyde reductase activity of AKR1C3.

Petra Haberzettl (University of Louisville, USA) reported on the interesting observation that aldose reductase (AKR1B1) protects against age-dependent insulin resistance in type 2 diabetes, which she concluded might be due to the fact that AKR1B1 metabolizes excess glucose via the polyol pathway.

Chi-Ching Hwang (Kaohsiung Medical University, Taiwan) explained the catalytic role of the flexible substrate binding loop in 3*α*-hydroxysteroid dehydrogenase/carbonyl reductase (3*α*-HSD/CR) from *Comamonas testosteroni*, which catalyzes the oxidation of androsterone to androstandione.

Adrian Lapthorn (University of Glasgow, UK) presented his results on the structure and function of the aldo-keto reductase AKR14A1 from *Escherichia coli* which shares strong structural similarities with tetrameric voltage-gated potassium channel β-subunit AKR6A2.

Trevor Penning (University of Pennsylvania, USA) described his groundbreaking work to surmount castration-resistant prostate cancer with AKR1C3 inhibitors. He and co-workers have developed three major classes of inhibitors based on non-steroidal anti-inflammatory drug analogues in which compounds were identified to have nano-molar potency for selective AKR1C3 inhibition.

Susanne Weber (Helmholtz Zentrum München, Germany) described a novel human gene locus, *AKR1B15*, which clusters with other members of the AKR1B sub-family, *AKR1B1* and *AKR1B10*, on chromosome 7. She found highest levels of *AKR1B15* mRNA in placenta and testis, and intermediate levels in prostate, ovary and skeletal muscle.

Paul-Georg Germann (Takeda Pharmaceutical, Europe) gave a perspective from industry on the translation from biochemical research into medicinal drugs. He suggested that the biomedical knowledge that is produced in basic research in many research institutions, including universities, should be exploited together with the industry to develop new therapeutic strategies.

Tea Lanišnik Rižner (University of Ljubljana, Slovenia) discussed the involvement of 17-ketosteroid reductases, AKR1C3 and 17β-HSD type 1, in estrogen biosynthesis in endometrial cancer. Based on her data, she concluded that in endometrial cancer, estrogen is formed from estrogen-sulphate and not from androstendione, suggesting that the sulphatase pathway has more importance for possible anti-estrogenic strategies than the aromatase pathway.

Mark Petrash (University of Colorado, USA) introduced an interesting concept where clues from Ayurveda led to a potential diabetes therapy directed at aldose reductase AKR1B1. Based on the observation that the Indian gooseberry (*Emblica officinalis*, amla) is used in traditional Indian medicine to minimize diabetic complications, and that these effects were consistent with AKR1B1 inhibition, he and co-workers identified AKR1B1 inhibitors in amla extracts.

Aruni Bhatnagar (University of Louisville, USA) addressed the function of aldose reductase (AKR1B1) in cardiac autophagy and reported on studies with wild-type and AKR1B1-null mice. He concluded that AKR1B1 prevents the activation of autophagic responses either by metabolizing glucose and/or removing aldehydes generated by oxidative changes that precede autophagy.

Oleg Barski (University of Louisville, USA) presented his provocative theory that aldehyde quenchers such as carnosine could serve as potential therapies for the prevention and treatment of atherosclerosis and inflammation. He and colleagues found that carnosine rapidly reacted with oxidized lipid-derived reactive aldehydes and that carnosine feeding inhibits atherogenesis by facilitating aldehyde removal and inhibiting ER stress.

## Conclusion and future perspectives

One of the strengths of this meeting was the laid-back, congenial atmosphere, which fostered the interaction between delegates. This not only created an environment where younger researchers could meet senior scientists, but also further facilitated the exchange of ideas and the start of collaborations. It was particularly gratifying to see so much new, unpublished data being presented. In summary, this year's Carbonyl Meeting continued the high standard of excellence set by previous conferences.

### Election of an executive committee and proposal to found a society

Attendance remained strong throughout the meeting, including the last session, which featured a lively debate on the future of the ‘International Meeting on he Enzymology and Molecular Biology of Carbonyl Metabolism’ (led by Edmund Maser). Several attendees addressed their concerns over budgetary and organisational issues, which finally resulted in the proposal to found a society. Seven senior scientists were nominated for the Executive Committee. It was agreed to find a name for the Society (together with an acronym) and select a president (chairperson), president-elect, treasurer and four additional persons within 2 years, i.e. before the meeting in 2014 which will be held in Philadelphia, USA.

### Social program

The highlights of the social program were a boat excursion on Lake Ploen on a Ploen cruiser followed by a guided walk through Princes' Island and a concert presented by the famous ‘Shanty Chorus’ during the gala dinner.

### Publication

Scientific contributions will appear as full peer-reviewed papers in a Special Issue of the international journal *Chemico*-*Biological Interactions*. The managing guest editor (Bryce Plapp) and four associate guest editors (Natasha Kedishvili, Tea Lanišnik-Rižner, Peter O'Brien and Edmund Maser) will work in the review process, which is planned to be completed in December 2012, such that the Special Issue of *Chemico*-*Biological Interactions* can appear in early 2013.

## Competing interests

The author declares that he has no competing interests.

